# The C-terminal transactivation domain of MITF interacts promiscuously with co-activator CBP/p300

**DOI:** 10.1038/s41598-023-43207-6

**Published:** 2023-09-26

**Authors:** Alexandra D. Brown, Kyle Lynch, David N. Langelaan

**Affiliations:** Department of Biochemistry and Molecular Biology, Dalhousie University, Halifax, NS B3H 4R2 Canada

**Keywords:** Biochemistry, NMR spectroscopy, Peptides

## Abstract

The microphthalmia-associated transcription factor (MITF) is one of four closely related members of the MiT/TFE family (TFEB, TFE3, TFEC) that regulate a wide range of cellular processes. MITF is a key regulator of melanocyte-associated genes, and essential to proper development of the melanocyte cell lineage. Abnormal MITF activity can contribute to the onset of several diseases including melanoma, where MITF is an amplified oncogene. To enhance transcription, MITF recruits the co-activator CREB-binding protein (CBP) and its homolog p300 to gene promoters, however the molecular determinants of their interaction are not yet fully understood. Here, we characterize the interactions between the C-terminal MITF transactivation domain and CBP/p300. Using NMR spectroscopy, protein pulldown assays, and isothermal titration calorimetry we determine the C-terminal region of MITF is intrinsically disordered and binds with high-affinity to both TAZ1 and TAZ2 of CBP/p300. Mutagenesis studies revealed two conserved motifs within MITF that are necessary for TAZ2 binding and critical for MITF-dependent transcription of a reporter gene. Finally, we observe the transactivation potential of the MITF C-terminal region is reliant on the N-terminal transactivation domain for function. Taken together, our study helps elucidate the molecular details of how MITF interacts with CBP/p300 through multiple redundant interactions that lend insight into MITF function in melanocytes and melanoma.

## Introduction

The microphthalmia transcription factor family (MiT/TFE) is comprised of four closely related DNA-binding proteins: transcription factor EB (TFEB), transcription factor E3 (TFE3), transcription factor EC (TFEC), and the microphthalmia-associated transcription factor (MITF)^1,2^. These transcription factors are highly evolutionarily conserved and homo/heterodimerize through a shared basic helix-loop-helix leucine zipper (bHLH) domain that recognizes and binds to E-BOX (CANNTG) and M-box (TCATGTG) binding sites in promoter regions of target genes^3^. The structural similarities between MiT transcription factors allows them to control numerous cellular processes including organelle biogenesis, energy homeostasis, and cell fate determination^4,5^.

Mutation to the *MITF* gene results in the failure of neural crest progenitor cells to properly differentiate, and is found in conditions including Wardenbuurg Type 2 and Tietz syndrome, which cause varying degrees of congenital hearing loss, abnormal retinal development, and pigmentation deficiencies^6,7^. Through the usage of alternative promoters and splicing of the first exon, the *MITF* gene generates various mRNA isoforms which differ based their amino termini^8^. While certain MITF isoforms are widely expressed (MITF-A, MITF-B and MITF-J)^9–11^, others are predominantly found in certain cell types including cardiomyocytes (MITF-H)^12^, cervical cells (MITF-CX)^13^, mast cells (MITF-E and MITF-MC)^14,15^, retinal pigment epithelium and osteoclasts (MITF-D)^16^. All MITF isoforms share identical functional domains to the shortest isoform (MITF-M), which is exclusive to melanocyte cells^11,17^. For simplicity in this manuscript, from here on reference to MITF refers to the melanocyte specific isoform MITF-M.

MITF is widely regarded as the master regulator of the melanocyte cell-lineage^18^. In melanocytes, MITF plays a critical role in regulating melanogenesis by directly targeting and controlling the expression of enzymes involved in melanin pigment synthesis (*TYR, TYRP1, DCT*)^19^. Aside from this function, MITF also modulates the expression of almost one hundred different melanocyte genes involved in cell cycle regulation (*CDK2, TBX2*), migration (*SNAI2, c-MET*), and survival (*BCL2, BIRC7, HIF1*⍺)^20,21^. Given the critical role of MITF in melanocyte biology, dysregulation of MITF activity is closely associated with the development and progression of the melanocyte-derived skin cancer melanoma^18^. MITF activity is linked to melanoma cell phenotype and behaviour, where it mediates switching from highly proliferative to highly invasive states promoting tumorigenesis and metastases of the disease^22^.

**Figure 1:**
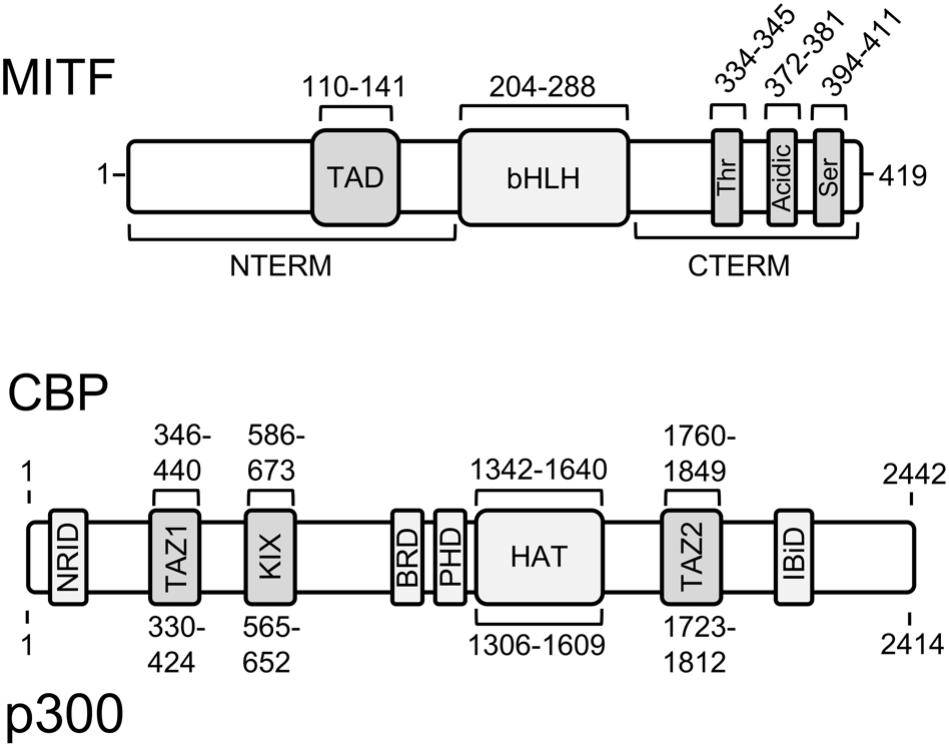
Schematic of MITF and CBP/p300 domains. MITF contains a basic helix-loop-helix DNA-binding domain (bHLH), N-terminal (NTERM) transactivation domain (TAD) and C-terminal region which contains threonine-rich (Thr), acidic, and serine-rich (Ser) regions. Domain boundaries are numbered based on the MITF-M isoform sequence. CBP/p300 has a catalytic histone acetyltransferase domain (HAT) and several protein-binding domains: nuclear receptor interaction domain (NRID), kinase inducible domain (KIX), IRF-3 binding domain (IBiD), and transcription adapter zinc finger domains (TAZ1 and TAZ2).

The ability of MITF to activate gene expression is in part attributed to its interactions with co-regulating proteins within transcriptional complexes. This includes the histone acetyltransferases CREB-binding protein and its homolog p300 (CBP/p300), which potentiate MITF transactivation by remodeling chromatin structure and making DNA more accessible to transcriptional machinery^23,24^. MITF recruits CBP/p300 through interactions facilitated by its N-terminal transactivation domain (TAD)^25^. While many studies have focused on the importance of MITF TAD, evidence has suggested that the C-terminal region of MITF also contains a transcriptionally competent domain, which when removed significantly impacts MITF-regulated transcription (Fig. 1)^26^. This raises the possibility that the MITF C-terminal region may function together with the N-terminal TAD to enhance MITFs ability to recruit transcriptional co-activators like CBP/p300. Despite the potential importance of the MITF C-terminal region, the structural details of this region or how it interacts with CBP/p300 are unclear.

In this study, we utilize a combination of nuclear magnetic spectroscopy (NMR), isothermal titration calorimetry (ITC), and protein pulldown assays to determine the structural features of the C-terminal region of MITF and define its interactions CBP/p300. We determine that the disordered MITF C-terminus interacts promiscuously with both transcriptional adaptor zinc finger domains (TAZ1 and TAZ2) of CBP/p300. Furthermore, we identify acidic and serine-rich motifs within the MITF C-terminal transactivation domain that are essential to maintain its high-affinity interaction with TAZ2, and when removed significantly reduce the ability of MITF to activate reporter gene transcription. Finally, we remark that the C-terminal MITF transactivation domain appears to work in cooperation with MITF TAD to active gene transcription.

## Results and discussion

### The MITF C-terminus is intrinsically disordered

**Figure 2:**
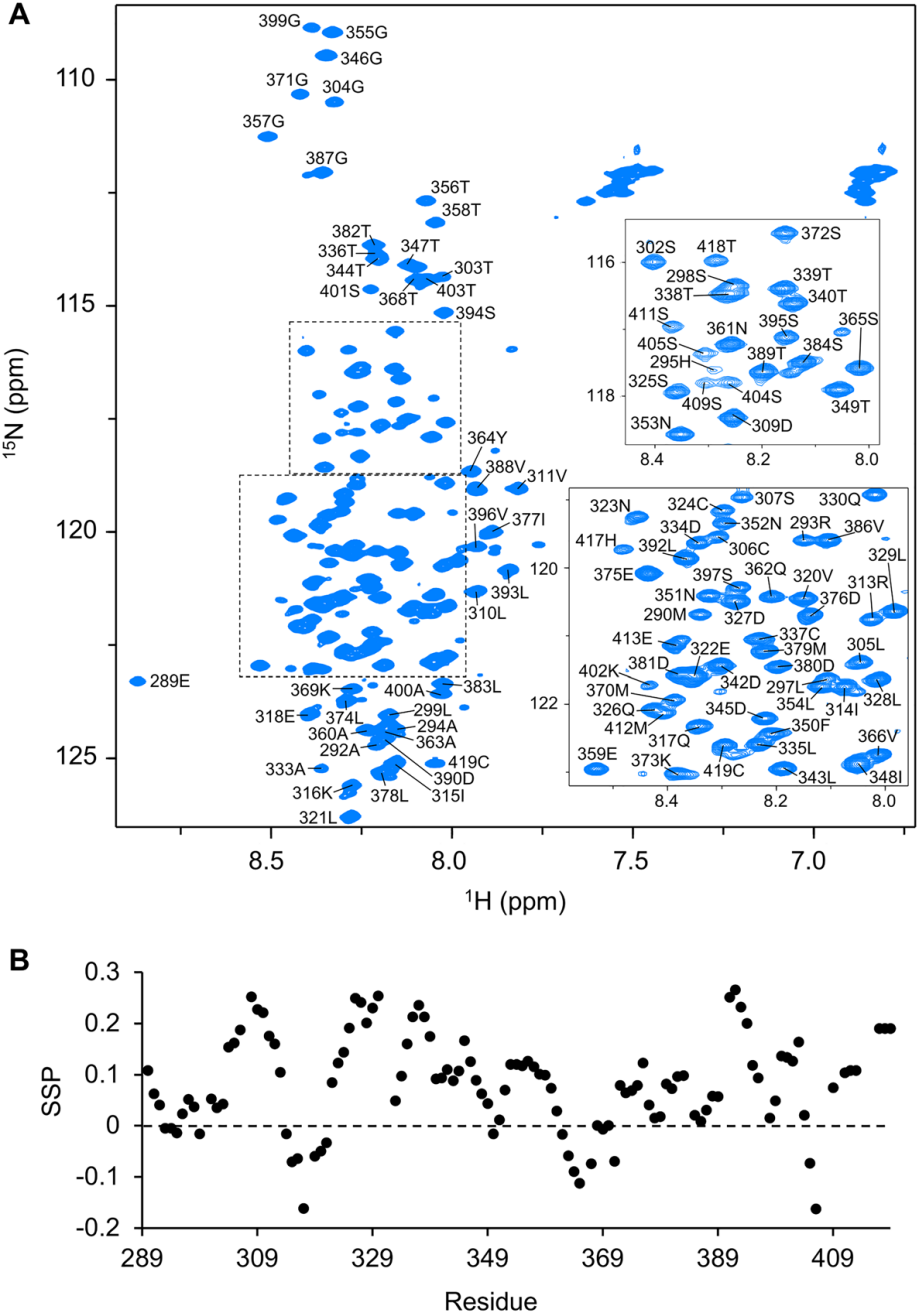
(**A**) ^1^H-^15^N-HSQC of ^13^C, ^15^N-labelled MITF_CTERM_, backbone amide resonance peaks are labelled based on their assigned residue. (**B**) Secondary structure propensity (SSP) values per residue of MITF_CTERM_ calculated from C⍺ and Cβ chemical shifts.

To analyze the structural features of the MITF C-terminal region, NMR experiments were collected on a ^13^C, ^15^N-labelled sample of MITF_CTERM_. The resulting ^1^H-^15^N HSQC spectrum was of high-quality with strong signal-to-noise intensities and the expected number of cross-peaks based on the number of non-proline MITF residues and (Fig. 2A). Utilizing standard triple resonance experiments, we achieved unambiguous assignment of 91% of backbone amide ^1^H and ^15^N resonances for MITF_CTERM_ (119/131 residues). This nearly complete assignment provided an excellent basis for both this study and future investigations of protein–protein interactions involving the MITF C-terminus.

The ^1^H-^15^N HSQC spectrum of MITF_CTERM_ had nearly all ^1^H chemical shifts fall within a narrow range of 0.6 ppm, a hallmark feature of intrinsically disordered proteins where both sequence biases and conformational plasticity contribute to the limited dispersion of proton chemical shifts^27,28^. We also calculated residue-specific secondary-structure propensity scores (SSP) based on the assigned backbone C⍺ and C_β_ chemical shifts, where a score of + 1 indicates the formation of a fully-formed ⍺-helix, − 1 a β-sheet, and values in between roughly corresponding to the fraction or propensity to adopt secondary structure at that position^29^. Along the entire sequence of MITF_CTERM_ residues had SSP scores ranging from − 0.2 to + 0.25, we calculate an overall total of 11.6% proclivity for forming ⍺-structure and 5.3% β-structure (Fig. 2B). This suggests that this region of MITF has significant random-coil propensity with minor tendency to form secondary structure, consistent with the small peak dispersion observed in the ^1^H-^15^N HSQC spectrum.

Intrinsically disordered proteins are abundant in the eukaryotic proteome, and particularly important to processes like signalling and regulation^30,31^. Disordered regions are prevalent amongst transactivation domains including those of the well-characterized p53 and c-Myc transcription factors^32,33^. Their conformational flexibility helps to facilitate binding of these transactivation domains to a variety of structurally diverse co-regulators within transcriptional complexes. These regions can undergo a disorder-to-order transition to fine-time their affinity for a particular binding partner, but many retain some structural ambiguity in their molecular recognition, termed ‘fuzzy’ complexes which can mediate highly specific but transient interactions necessary to adapt to changing cellular stimuli^34,35^.

### The C-terminal region of MITF binds both TAZ domains of CBP/p300

**Figure 3:**
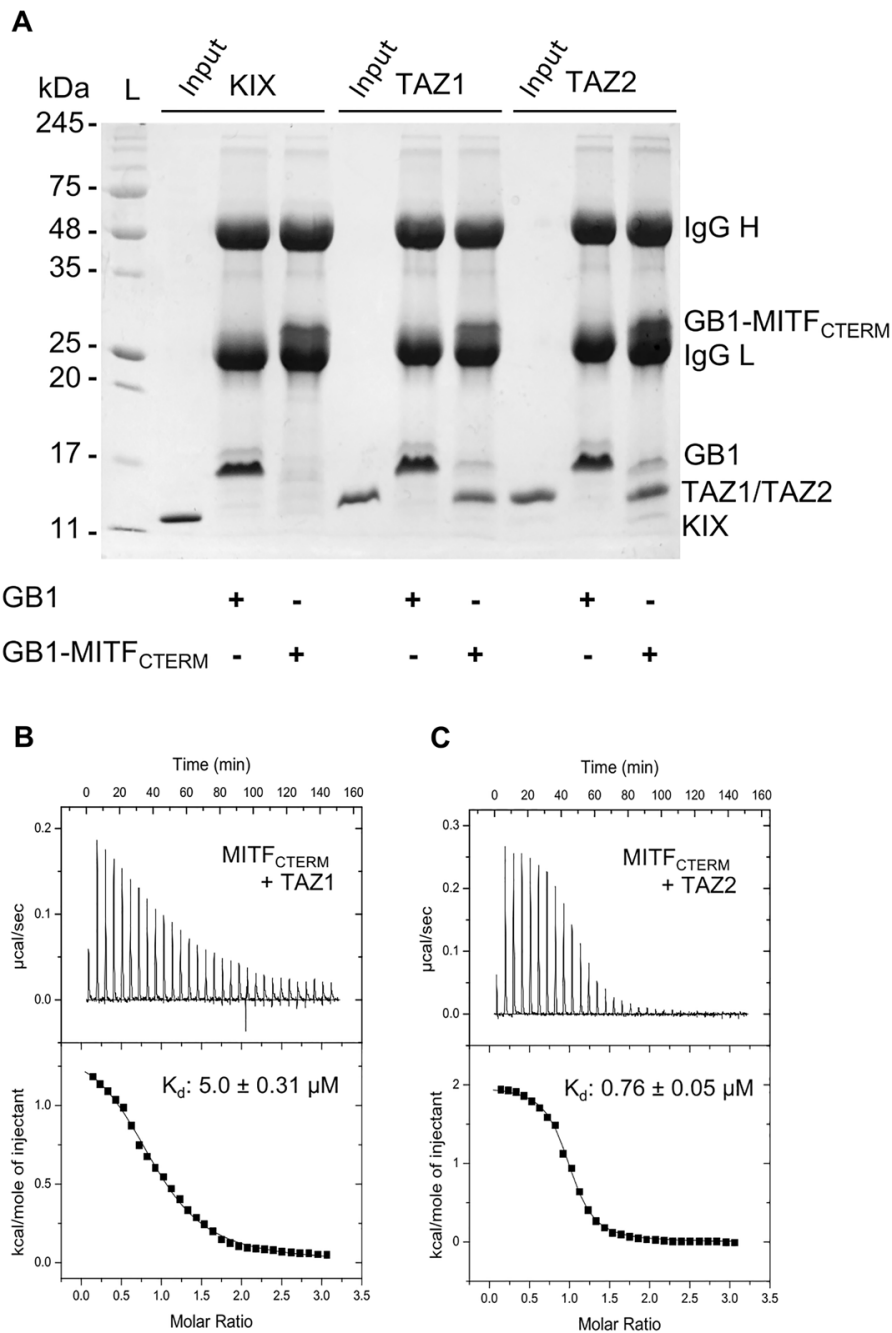
(**A**) Coomassie-stained SDS-PAGE analysis of the total amount of KIX, TAZ1, or TAZ2 pulled down by immobilized GB1 or GB1-MITF_CTERM._ Lanes 2, 5, and 8 show migration of each isolated CBP/p300 domain and represents 10% of total pulldown input. The migration of immunoglobulin heavy and light chains (IgG H and IgG L), GB1, GB1-MITF_CTERM_, KIX, TAZ1, and TAZ2 are denoted. Duplicate uncropped gels are shown in Fig. S3. (**B**) ITC thermogram following titration of TAZ1 (400 µM) or (**C**) TAZ2 (400 µM) into MITF_CTERM_ (30 µM). Data were fit to one-site binding curves, determining dissociation constants (K_d_) of 5.0 ± 0.31 µM and 0.76 ± 0.05 µM for the interaction of MITF_CTERM_ with TAZ1 and TAZ2, respectively).

CBP/p300 are large multi-domain proteins that play an important role connecting numerous independent cell-signalling pathways through interactions with hundreds of regulators from all major transcription factor families^36,37^. Within transcriptional complexes, CBP/p300 provide a scaffold to coordinate various sequence-specific transcription factors through interactions with their multiple conserved protein-binding domains. These include the kinase inducible domain (KIX) and transcriptional adaptor zinc finger domains (TAZ1 and TAZ2) (Fig. 1B) which facilitate binding of CBP/p300 to transcription factors including p53, E2A, and STAT1/2^38–41^. To test if any of these domains interact with the MITF C-terminus, we performed a protein pulldown assay where GB1-MITF_CTERM_ was immobilized and incubated with purified KIX, TAZ1, or TAZ2. SDS-PAGE analysis of total protein following these experiments indicated that GB1-MITF_CTERM_ did not interact with the KIX domain of CBP/p300 (Fig. 3A), while it did interact with both the TAZ1 and TAZ2 domains.

To quantitatively assess these interactions we next performed isothermal titration calorimetry, where isolated TAZ1 or TAZ2 was injected into a reaction cell containing purified MITF_CTERM_. The resulting thermograms indicated that MITF_CTERM_ interacts with TAZ1 with a binding affinity (K_d_) of 5.0 ± 0.31 µM, and with TAZ2 with a ~ 6 × higher affinity (K_d_ = 0.76 ± 0.05 µM) (Fig. 3B,C). These findings correlate with our protein pulldown data which suggested that MITF binds to both domains. They are also comparable to the MITF N-terminal TAD which binds TAZ1 with a K_d_ ~ 6.7 µM and TAZ2 with a K_d_ ~ 1.2 µM, and to the p53 transactivation domain which similarly binds TAZ1 with a K_d_ ~ 0.9 µM and TAZ2 with a K_d_ ~ 26 nM^25,42^. Given that the availability of transcriptional co-regulators can directly influence MITF activity, and that CBP/p300 is a hub for a multitude of transcription factor interactions across many different signalling cascades, the ability of MITF to bind multiple CBP/p300 domains may help with its potential to re-direct the co-activator from other signaling pathways to enhance its own function.

### Identification of functionally important C-terminal MITF residues

**Figure 4:**
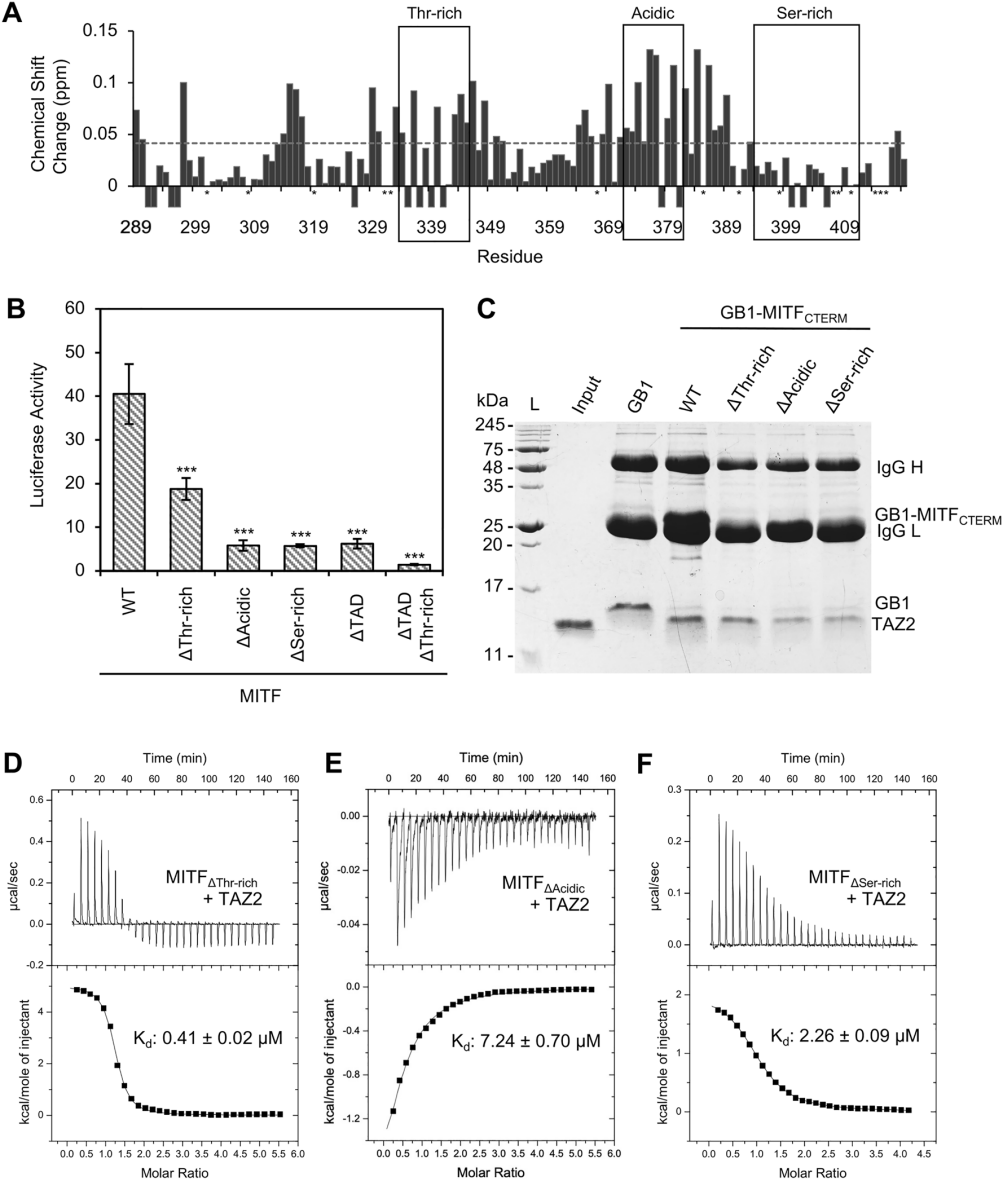
(**A**) Chemical shift perturbations (∆δ = [(0.17Δδ_N_)^2^ + (Δδ_HN_)^2^]^1/2^) of backbone resonances per residue of ^15^N-labelled MITF_CTERM_ (100 µM) upon the addition of TAZ2 (300 μM). Negative values indicate disappearance of a resonance peak, and * denotes a proline or unassigned resonance. Average chemical shift change (0.04 ppm) is shown as dashed line, boxes denote regions chosen for mutation. (**B**) Average luciferase activation of MITF constructs, reported as fold activation of pcDNA3.1 negative control. Error bars represent standard error, statistical analysis performed using one-way ANOVA followed by Dunnett’s multiple comparison test (***p ≤ 0.001). (**C**) Coomassie-stained SDS-PAGE analysis of total TAZ2 pulled down by immobilized GB1-MITF_CTERM_ or GB1-tagged MITF mutant constructs MITF_ΔThr-rich_ (Δ334–345)_,_ MITF_ΔAcidic_ (Δ372–381)_,_ and MITF_ΔSer-rich_ (Δ394–411). Lane 2 shows migration of isolated TAZ2 domain and represents 10% total pulldown input. The migration of immunoglobulin heavy and light chains (IgG H and IgG L), GB1, GB1-MITF_CTERM_, and TAZ2 are denoted. Uncropped images of duplicate experiments are found in Fig. S4. (**D**–**F**) ITC thermograms of MITF_ΔThr-rich_ (16 µM), MITF_ΔAcidic_ (17 µM), and MITF_ΔSer-rich_ (22 µM), following titration with TAZ2 (400 µM) each fit to a one-site binding curve (K_d_ = 0.41 ± 0.02 µM, 7.24 ± 0.70 µM, and 2.26 ± 0.09 µM, respectively).

We decided to further characterize how TAZ2 binds MITF_CTERM_, given its higher affinity to MITFC_TERM_ than TAZ1. To determine which residues of the MITF C-terminus were involved in binding TAZ2, a ^15^N-labelled MITF_CTERM_ sample was prepared and sequential ^1^H-^15^N-HSQC spectra were collected upon titration with unlabelled TAZ2 (Fig. S1). Once saturated with TAZ2, chemical shift changes (Δδ) experienced by MITF_CTERM_ backbone resonance peaks were then calculated (Fig. 4A). From these calculations it was determined that most MITF_CTERM_ residues experienced only minor changes in chemical shift (< 0.15 ppm). While there appears to be a higher number of perturbations located between the regions L329-I348 and K369-T389, others resonances throughout the protein disappeared from the spectra entirely. This disappearance of resonance peaks may be caused by intermediate chemical exchange leading to line broadening and signifies that these residues could be involved in the intermolecular interaction with TAZ2^43,44^.

The transactivation domain architecture of MiT/TFE transcription factors include either acidic or serine/threonine/proline-rich regions that can work synergistically with one another^45^. For example, TFE3 the closest relative to MITF, contains two activation domains, a C-terminal proline-rich TAD that both contributes to transactivation and potentiates the function of its N-terminal acidic TAD^46,47^. Given the homology between MITF and TFE3 (58% sequence identity), it is reasonable to believe the MITF C-terminal transactivation domain may function in a similar synergistic fashion to enhance the activity of the MITF N-terminal transactivation domain. Taking into consideration sequence homology and our NMR perturbation studies, three mutants were generated to remove a threonine-rich region from residues D334-D345, an acidic region from residues S372-D381, and a serine-rich region from residues S394-S411 (Figs. 4A, S2). To determine if any of these regions are important for transactivation, a luciferase-based transcriptional assay was performed where HEK 293A cells were transfected with wildtype or deletion mutants of MITF and a reporter containing 7 E-BOX binding sites upstream a luciferase gene. Normalized luminescence values were determined, and MITF was seen to activate transcription 40-fold higher than negative controls. Comparatively, deletion of the threonine-rich region (Δ334–345) reduced the transcriptional ability of MITF by half while deletion of the acidic (Δ372–381) or serine-rich (Δ394–411) regions diminished activation eight-times less than wild-type (Fig. 4B). These findings coincide with previous studies which have shown that removal of the entire MITF C-terminal region (residues 324–419) notably decreases MITFs ability to activate transcription of the tyrosinase gene, but a region containing only the threonine-rich motif (residues 324–369) still retains modest activation potential when fused to a GAL4 DNA-binding domain^48^.

To test if this transcriptional activity was functioning independently, redundantly, or cooperatively with the MITF N-terminal transactivation domain (TAD), ΔTAD mutants were generated (removing residues 110–141) from MITF and MITF_ΔThr-rich_ upon which any transcriptional activity occurred by those constructs was ablated (Fig. 4B). Given that the removal of both the C-terminal threonine-rich region and MITF TAD were required for complete loss of transactivation, this suggests a cooperative mechanism similar to TFE3, where both domains are necessary for MITF to be fully functional^46^. Given that all isoforms of MITF have the same C-terminal sequences, we anticipate that our in-vitro studies of MITF_CTERM_ are relevant to all isoforms of MITF. However, the degree of cooperativity observed with MITF_TAD_ will likely be isoform specific since MITF isoforms have differing N-termini.

### C-terminal MITF function correlates to TAZ2 binding

The reduced transactivation potential observed for the MITF mutants could be due to several factors beyond weaking the association with CBP/p300, such as changes in expression level or subcellular localization. To directly test which regions of the MITF_CTERM_ have some ability to bind TAZ2, we performed an in vitro protein pulldown assay where GB1-tagged MITF_CTERM_ or deletion mutants were tested for their ability to interact with purified TAZ2 (Fig. 4C). While all mutants retained some residual ability to interact with TAZ2, mutants which removed either the acidic (Δ372–381), or serine-rich (Δ394–411) regions greatly reduced the amount of TAZ2 that was retained in comparison to the wild-type domain. Consistent with these results, ITC studies revealed that deletion of the threonine-rich region (Δ334–345) did not have any significant impact on the affinity of the MITF_CTERM_ for TAZ2 (K_d_ 0.41 ± 0.02 µM; Fig. 4D), whereas deletion of the acidic region, or serine-rich region impacted TAZ2 binding by tenfold and threefold, respectively (K_d_ = 7.24 ± 0.70 µM and 2.26 ± 0.09 µM; Fig. 4E,F).

Interestingly, removal of the threonine-rich region, which had the smallest impact on MITF transcription activity and TAZ2 binding, is the least conserved amongst all MiT family members (Fig. S2). On the other hand, the acidic region shares considerable homology with conserved aspartic acid and glutamic acid residues at positions E375, D380, and D381. Removal of this region from MITF_CTERM_ caused the greatest disruption of TAZ2 binding, likely due to the loss of these negatively charged residues which would mediate interactions with the highly electropositive surface of TAZ2.

The serine-rich region contains eight evolutionary conserved serine residues present in all other MiT family members including S397, S401, S405 which are known phosphorylation targets of glycogen synthase kinase 3, and S409 a previously identified target of AKT and RSK mediated phosphorylation^49–51^. Evidence for such post-translational modifications may influence the subcellular localization and electrostatic interactions between MITF and CBP/p300, both of which would impact transactivation potential of MITF.

Overall, we have determined that the MITF_CTERM_ is intrinsically disordered and interacts with both the TAZ1 and TAZ2 domains of CBP/p300. We have identified an acidic and a serine-rich motif within the MITF C-terminal transactivation domain that are essential to both MITF transcriptional function and TAZ2-recruitment of CBP/p300. Finally, we observe that the C-terminal region of MITF cooperates with the N-terminal transactivation domain of MITF to active gene transcription. These finding advance overall understanding of MITF-mediated transcription and are fundamental to our knowledge of co-activator recruitment in gene regulation.

## Materials and methods

### Plasmid constructs

The cDNA of MITF isoform M (Genbank: Accession no. BC065243.1) was purchased from ThermoFisher Inc. and a region encoding for C-terminal MITF-M (residues 289–419, CTERM, corresponding to residues 396–526 in the canonical MITF sequence) was cloned into restriction sites *BglII/SalI* of a pET21b derived vector modified to include a hexahistidine tag, the B1 domain of *Streptococcal* protein G (GB1), and a tobacco-etch virus recognition site, to derive pGB1-MITF_CTERM_. MITF variants: MITF_ΔThr-rich_ MITF_ΔAcidic_ and MITF_ΔSer-rich_ (Δ334–354, Δ372–381 and Δ372–381, respectively, and corresponding to Δ441–452, Δ479–488 and Δ501–518 in the canonical sequence) were produced by site-directed mutagenesis of pGB1-MITF_CTERM_ using in vivo assembly cloning where deletions were incorporated into primers^52^. Constructs for the domains of CBP/p300 (TAZ1, TAZ2, and KIX) were as previously described^39^. For transcriptional assays, a pGL3 luciferase reporter plasmid was purchased from BioBasic Inc. and modified to include 7 E-BOX (CACGTG) binding sites upstream a luciferase reporter gene (p7xE-BOX). Constructs for mammalian expression of full-length MITF were provided by Yardena Samuels (Addgene plasmid #31151; pCMV-MITF-WT), and mutant constructs were produced from full-length pCMV-MITF-WT using Q5 site-directed mutagenesis (New England Biolabs). The validity of all constructs was confirmed using DNA sequencing.

### Protein expression and purification

MITF constructs** (**GB1-MITF_CTERM_, GB1-MITF_ΔThr-rich_, GB1-MITF_ΔAcidic,_ GB1-MITF_ΔSer-rich_) were expressed in *Escherichia coli* BL21 (DE3). Cells were grown in Lysogeny Broth under ampicillin selection until a mid-log phase and optical density of 0.6–0.8 was reached and then expression was induced with the addition of 0.5 mM isopropyl-β-d-thiogalactopyranoside at 37 °C for 4 h. Isotopically labelled samples were produced by cells grown in ^15^N- or ^13^C,^15^N enriched M9 minimal media^53^. Cells (1 L) were harvested by centrifugation and resuspended in 30 mL lysis buffer (20 mM Tris–HCl pH 8, 250 mM NaCl, 8 M urea), sonicated for 3 min, centrifuged in SS-34 rotor (Sorvall) at 14,500 rpm to clarify lysate which was then applied to a Ni–NTA affinity column (Cytiva) and washed with lysis buffer containing 10 mM imidazole. Proteins were refolded on column with the addition of native buffer (20 mM Tris–HCL pH 8, 250 mM NaCl, 5 mM βME) and eluted with native buffer containing 300 mM imidazole. Refolded protein was then dialyzed overnight at 4 °C into dialysis buffer (20 mM Tris–HCl pH 8, 50 mM NaCl, 5 mM βME) in the presence of TEV protease (150 µg). The GB1 affinity tag was removed using another round of Ni–NTA chromatography, and the flowthrough containing cleaved MITF constructs was further purified using Q-Sepharose anion exchange column (Cytiva) equilibrated with IEC buffer (20 mM Tris–HCl pH 8, 5 mM βME). The column was washed with IEC buffer containing 50 mM NaCl, and the final protein was eluted using IEC buffer containing 500 mM NaCl. Expression of CBP/p300 domains (TAZ1, TAZ2, and KIX) were as previously described^39^. Protein fractions were analyzed by SDS-PAGE and quantified using UV–Vis spectroscopy.

### NMR spectroscopy

A ^13^C,^15^N-labelled sample of MITF_CTERM_ (700 μM) was prepared in NMR buffer (20 mM MES pH 6, 25 mM NaCl, 5 mM DTT, 5% D_2_O). Backbone resonances were then assigned by measuring ^1^H-^15^N HSQC, HNCO, HN(CA)CO, CBCACONH, and HNCACB spectra, and following resonance assignment secondary structure propensity (SSP) was calculated based on C⍺ and Cβ chemical shifts^29^. All NMR experiments were collected at 35 °C on a Bruker Avance III 700 MHz spectrometer at the National Research Council Institute for Marine Biosciences (Halifax, NS). Data was processed using NMRPipe and assigned using CcpNmr Analysis^54,55^.

Chemical shift assignments for MITF_CTERM_ have been deposited at the Biological Magnetic Resonance Data Bank under accession no. 51763. To assess binding between MITF _CTERM_ and TAZ2, a sample of ^15^N-labelled MITF_CTERM_ (100 μM) was prepared in NMR buffer and ^1^H-^15^N HSQC spectra were collected in the absence or presence of increasing amounts of unlabelled TAZ2 (up to 300 μM). Chemical shift perturbations (∆δ) of MITF resonances were then measured upon addition of TAZ2 (∆δ = [(0.17Δδ_N_)^2^ + (Δδ_HN_)^2^]^1/2^)^56^.

### Pulldown assays

Indicated GB1-tagged fusion proteins or GB1 as a control (20 nmoles), were incubated with 20 µL IgG agarose beads (Cytiva) in binding buffer (20 mM Tris–HCl pH 8, 50 mM NaCl, 5 mM βME, 10 μM ZnCl_2_) and rotated end-over-end at room temperature for 15 min. Beads were then collected by microcentrifugation (1 min at 2000 rpm), washed two times with binding buffer, before an equimolar amount of TAZ1 or TAZ2 was added and rotated for 30 min. The protein complexes bound to the beads were washed three more times with binding buffer, and the total protein present was then analyzed by SDS-PAGE.

### Isothermal titration calorimetry

All ITC experiments were performed in buffer containing 20 mM TRIS pH 8, 25 mM NaCl, 5 mM βME, 10 µM ZnCl_2_. A total concentration of 400 µM TAZ1 or TAZ2 was loaded into the ITC syringe and incrementally injected into the sample cell containing 16–30 µM of purified MITF construct. Experiments were carried out using a VP-ITC Microcalorimeter with the following parameters: 30 injections of 10 µL with 300 s equilibration intervals, reference power 20 µCal/s, and stirring speed of 300 at 30 °C. Thermograms were fit assuming a single-binding site using MicroCal Origin 7.0 software and each experiment was performed in duplicate.

### Transcriptional assays

For all luciferase-based transcriptional assays, HEK 293A cells were cultured in DMEM supplemented with 10% FBS and incubated at 37 °C with 5% CO_2_. Cells were seeded 24-h prior to transfection into 24-well plates and transfected with jetPRIME reagent. To each well, 0.5 µg of plasmid was added including: 0.35 µg p7xE-BOX reporter, 0.05 µg pCMV-Renilla, and 0.1 µg of pCMV-MITF expression plasmid. Cells were harvested 24 h after transfection at which point luminescence was measured from cell lysate using a Dual-Luciferase Reporter Assay System (Promega). Each experiment is a representation of *Renilla*-normalized luminescence from triplicate experiments and shown as fold activation relative to pcDNA3.1 negative control. Statistical significance was measured using one-way AVONA and Dunnett’s multiple comparison test compared to pCMV-MITF-WT.

### Supplementary Information

Supplementary Figures.

## Data Availability

The chemical shifts for MITF_CTERM_ have been deposited into BioMagResBank under accession code 51763.

## References

[1] Kim S, Song H-S, Yu J, Kim Y-M (2021). MiT family transcriptional factors in immune cell functions. Mol. Cells.

[2] Goding CR, Arnheiter H (2019). MITF—the first 25 years. Genes Dev..

[3] Hemesath TJ (1994). microphthalmia, a critical factor in melanocyte development, defines a discrete transcription factor family. Genes Dev..

[4] La Spina, M. *et al.* MiT/TFE family of transcription factors: An evolutionary perspective. *Front. Cell Dev. Biol.* **8**, (2021). 10.3389/fcell.2020.609683 PMC7815692 33490073

[5] Ballesteros-Álvarez J (2020). MITF and TFEB cross-regulation in melanoma cells. PLOS ONE.

[6] Tassabehji M, Newton VE, Read AP (1994). Waardenburg syndrome type 2 caused by mutations in the human microphthalmia (MITF) gene. Nat. Genet..

[7] Amiel J, Watkin PM, Tassabehji M, Read AP, Winter RM (1998). Mutation of the MITF gene in albinism-deafness syndrome (Tietz syndrome). Clin. Dysmorphol..

[8] Shibahara S (2001). Microphthalmia-associated transcription factor (MITF): Multiplicity in structure, function, and regulation. J. Investig. Dermatol. Symp. Proc..

[9] Amae S (1998). Identification of a novel isoform of microphthalmia-associated transcription factor that is enriched in retinal pigment epithelium. Biochem. Biophys. Res. Commun..

[10] Udono, T. *et al.* Structural organization of the human microphthalmia-associated transcription factor gene containing four alternative promoters11The nucleotide sequence data shown have been deposited in the GSDB/DDBJ/EMBL/NCBI DNA databases with the following accession numbers: AB032357 for exon 1A and its flanking regions, AB032358 for exon 1H and its flanking regions, AB032359 for exon 1B and its flanking regions and AB009608 for the 5′-flanking region of exon 1M of the MITF gene. *Biochim. Biophys. Acta (BBA) Gene Struct. Expression* **1491**, 205–219 (2000). 10.1016/s0167-4781(00)00051-8 10760582

[11] Bharti K, Liu W, Csermely T, Bertuzzi S, Arnheiter H (2008). Alternative promoter use in eye development: Complex role and regulation of the transcription factor MITF. Development.

[12] Tshori S (2006). Transcription factor MITF regulates cardiac growth and hypertrophy. J. Clin. Invest..

[13] Li X-H (2010). A novel isoform of microphthalmia-associated transcription factor inhibits IL-8 gene expression in human cervical stromal cells. Mol. Endocrinol..

[14] Oboki K, Morii E, Kataoka TR, Jippo T, Kitamura Y (2002). Isoforms of mi transcription factor preferentially expressed in cultured mast cells of mice. Biochem. Biophys. Res. Commun..

[15] Takemoto CM, Yoon Y-J, Fisher DE (2002). The identification and functional characterization of a novel mast cell isoform of the microphthalmia-associated transcription factor. J. Biol. Chem..

[16] Takeda K (2002). Mitf-D, a newly identified isoform, expressed in the retinal pigment epithelium and monocyte-lineage cells affected by Mitf mutations. Biochim. Biophys. Acta.

[17] Steingrímsson E, Copeland NG, Jenkins NA (2004). Melanocytes and the microphthalmia transcription factor network. Annu. Rev. Genet..

[18] Kawakami A, Fisher DE (2017). The master role of microphthalmia-associated transcription factor in melanocyte and melanoma biology. Lab. Invest..

[19] Yu F (2021). Mitf involved in innate immunity by activating tyrosinase-mediated melanin synthesis in Pteria penguin. Front. Immunol..

[20] Hoek KS (2008). Novel MITF targets identified using a two-step DNA microarray strategy. Pigment Cell Melanoma Res..

[21] Hartman ML, Czyz M (2015). MITF in melanoma: Mechanisms behind its expression and activity. Cell. Mol. Life Sci..

[22] Carreira S (2006). Mitf regulation of Dia1 controls melanoma proliferation and invasiveness. Genes Dev..

[23] Ogryzko VV, Schiltz RL, Russanova V, Howard BH, Nakatani Y (1996). The transcriptional coactivators p300 and CBP are histone acetyltransferases. Cell.

[24] Sato S (1997). CBP/p300 as a co-factor for the Microphthalmia transcription factor. Oncogene.

[25] Brown AD (2023). Structural basis of CBP/p300 recruitment by the microphthalmia-associated transcription factor.

[26] Vachtenheim J, Drdová B (2004). A dominant negative mutant of microphthalmia transcription factor (MITF) lacking two transactivation domains suppresses transcription mediated by wild type MITF and a hyperactive MITF derivative. Pigment Cell Res..

[27] Dyson HJ (2016). Making sense of intrinsically disordered proteins. Biophys. J..

[28] Chhabra, S. *et al.*^15^ N detection harnesses the slow relaxation property of nitrogen: Delivering enhanced resolution for intrinsically disordered proteins. *Proc. Natl. Acad. Sci. U.S.A.* **115**, (2018). 10.1073/pnas.1717560115 PMC5828609 29432148

[29] Marsh JA, Singh VK, Jia Z, Forman-Kay JD (2006). Sensitivity of secondary structure propensities to sequence differences between alpha- and gamma-synuclein: Implications for fibrillation. Protein Sci..

[30] Dunker AK, Obradovic Z, Romero P, Garner EC, Brown CJ (2000). Intrinsic protein disorder in complete genomes. Genome Inf. Ser. Workshop Genome Inf..

[31] Babu MM (2016). The contribution of intrinsically disordered regions to protein function, cellular complexity, and human disease. Biochem. Soc. Trans..

[32] Raj N, Attardi LD (2017). The transactivation domains of the p53 protein. Cold Spring Harb. Perspect. Med..

[33] Andresen C (2012). Transient structure and dynamics in the disordered c-Myc transactivation domain affect Bin1 binding. Nucleic Acids Res..

[34] Brodsky S, Jana T, Barkai N (2021). Order through disorder: The role of intrinsically disordered regions in transcription factor binding specificity. Curr. Opin. Struct. Biol..

[35] Sharma R, Raduly Z, Miskei M, Fuxreiter M (2015). Fuzzy complexes: Specific binding without complete folding. FEBS Lett..

[36] Chan HM, La Thangue NB (2001). p300/CBP proteins: HATs for transcriptional bridges and scaffolds. J. Cell Sci..

[37] Philip P (2015). CBP binding outside of promoters and enhancers in Drosophila melanogaster. Epigenet. Chromatin.

[38] Dyson HJ, Wright PE (2016). Role of intrinsic protein disorder in the function and Interactions of the transcriptional coactivators CREB-binding protein (CBP) and p300. J. Biol. Chem..

[39] Lochhead, M. R. *et al.* Structural insights into TAZ2 domain-mediated CBP/p300 recruitment by transactivation domain 1 of the lymphopoietic transcription factor E2A. *J. Biol. Chem.* jbc.RA119.011078 (2020). 10.1074/jbc.RA119.011078. 10.1074/jbc.RA119.011078 PMC7105314 32098872

[40] Lee CW, Martinez-Yamout MA, Dyson HJ, Wright PE (2010). Structure of the p53 transactivation domain in complex with the nuclear coactivator binding domain of CBP. Biochemistry.

[41] Wojciak JM, Martinez-Yamout MA, Dyson HJ, Wright PE (2009). Structural basis for recruitment of CBP/p300 coactivators by STAT1 and STAT2 transactivation domains. EMBO J..

[42] Ferreon JC (2009). Cooperative regulation of p53 by modulation of ternary complex formation with CBP/p300 and HDM2. Proc. Natl. Acad. Sci..

[43] Maity, S., Gundampati, R. K. & Suresh Kumar, T. K. NMR methods to characterize protein-ligand interactions. *Nat. Prod. Commun.* **14**, 1934578X19849296 (2019).

[44] Teilum K, Kunze MBA, Erlendsson S, Kragelund BB (2017). (S)Pinning down protein interactions by NMR. Protein Sci..

[45] Chin K-C, Li GG-X, Ting JP-Y (1997). Importance of acidic, proline/serine/threonine-rich, and GTPbinding regions in the major histocompatibility complex class II transactivator: Generation of transdominant-negative mutants. Proc. Natl. Acad. Sci..

[46] Artandi SE, Merrell K, Avitahl N, Wong KK, Calame K (1995). TFE3 contains two activation domains, one acidic and the other proline-rich, that synergistically activate transcription. Nucleic Acids Res.

[47] Kawata Y (2002). bcn-1 element-dependent activation of the Laminin γ1 chain gene by the cooperative action of transcription factor E3 (TFE3) and Smad proteins. J. Biol. Chem..

[48] Takeda K (2000). Ser298 of MITF, a mutation site in Waardenburg syndrome type 2, is a phosphorylation site with functional significance. Human Mol. Genet..

[49] Ploper D (2015). MITF drives endolysosomal biogenesis and potentiates Wnt signaling in melanoma cells. Proc. Natl. Acad. Sci..

[50] Wang C (2016). Phosphorylation of MITF by AKT affects its downstream targets and causes TP53-dependent cell senescence. Int. J. Biochem. Cell Biol..

[51] Wu M (2000). c-Kit triggers dual phosphorylations, which couple activation and degradation of the essential melanocyte factor Mi. Genes Dev..

[52] García-Nafría J, Watson JF, Greger IH (2016). IVA cloning: A single-tube universal cloning system exploiting bacterial in vivo assembly. Sci. Rep..

[53] Studier FW (2005). Protein production by auto-induction in high density shaking cultures. Protein Exp. Purif..

[54] Delaglio, F. *et al.* NMRPipe: A multidimensional spectral processing system based on UNIX pipes. *J. Biomol. NMR* **6**, 1 (1995). 10.1007/BF00197809 8520220

[55] Vranken WF (2005). The CCPN data model for NMR spectroscopy: Development of a software pipeline. Proteins Struct. Funct. Bioinf..

[56] Chitayat S, Kanelis V, Koschinsky ML, Smith SP (2007). Nuclear magnetic resonance (NMR) solution structure, dynamics, and binding properties of the Kringle IV type 8 module of apolipoprotein(a). Biochemistry.

