# Supplementary material for: The C-terminal transactivation domain of MITF interacts promiscuously with co-activator CBP/p300
Source: Sci Rep. 2023 Sep 26;13:16094. doi: 10.1038/s41598-023-43207-6 (PMC10522771; doi:10.1038/s41598-023-43207-6)
Supplement: Supplementary file 1 — Supplementary Figures. [file 41598_2023_43207_MOESM1_ESM.pdf]

Supplementary material for:

The C-terminal transactivation domain of MITF interacts promiscuously with co-activator CBP/p300

Alexandra D. Brown, Kyle Lynch, and David N. Langelaan*

Department of Biochemistry & Molecular Biology, Dalhousie University, Halifax, B3H 4R2, Canada

*To whom correspondence should be addressed: David N. Langelaan, Department of Biochemistry & Molecular Biology, Dalhousie University, Halifax, NS, B3H 4R2, Canada; david.langelaan@dal.ca

Contents:

Supplementary figures S1-S4

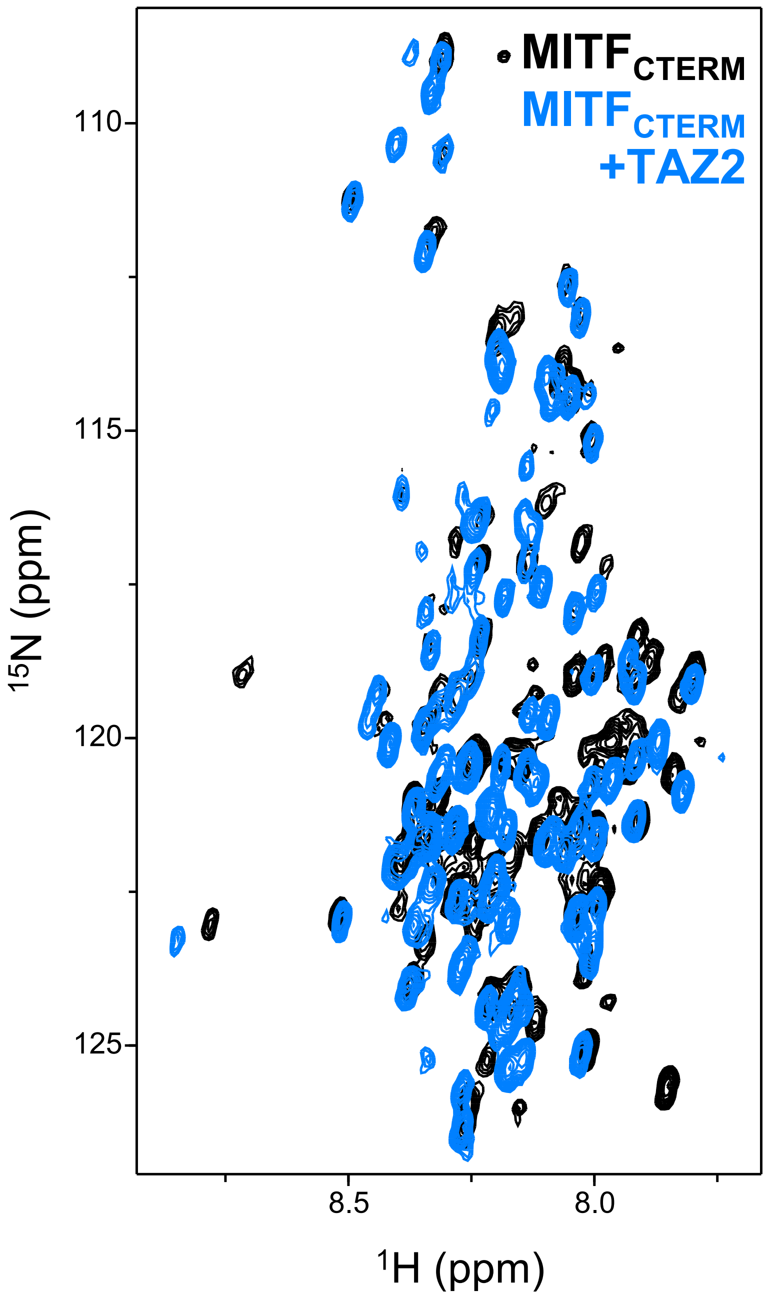

Figure S1. ^{1}H-^{15}N HSQC of 100 μM ^{15}N-labelled $MITF_{CTERM}$ in the absence (black) and presence (blue) of 300 μM unlabeled TAZ1.

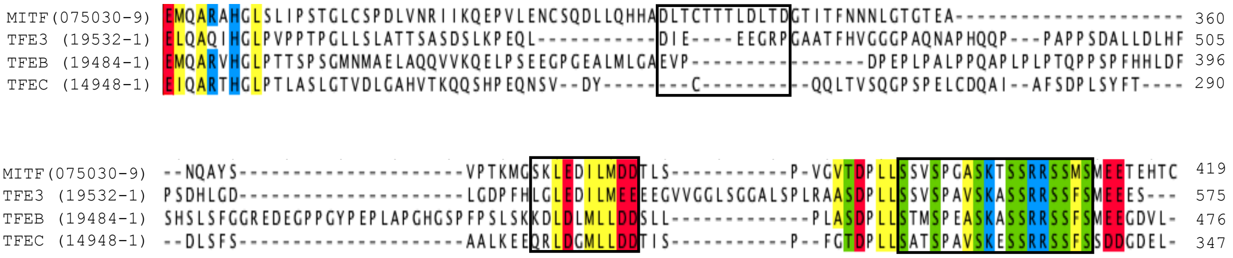

Figure S2. Multiple protein sequence alignment of MITF Isoform M and other MiT family members (TFE3, TFEB, TFEC) showing conservation of the C-terminal region. Residues with amino acid similarity (≥75%) are indicated by coloured shading, hydrophobic (yellow), positive (blue), negative (red), or polar uncharged (green). Boxes denote mutants used in this study labels correspond to positions in native amino acid sequences.

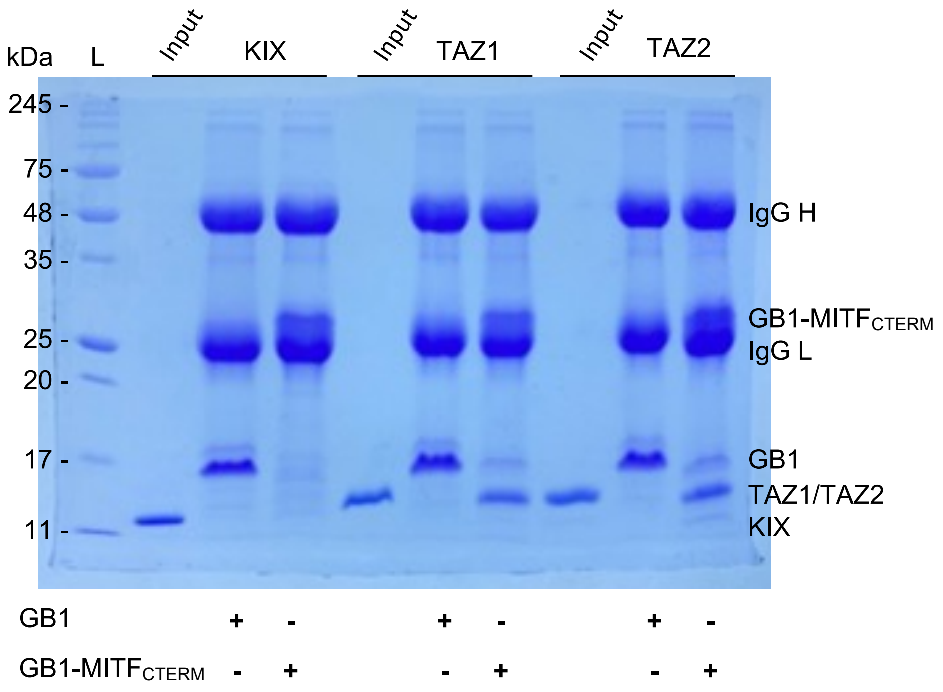

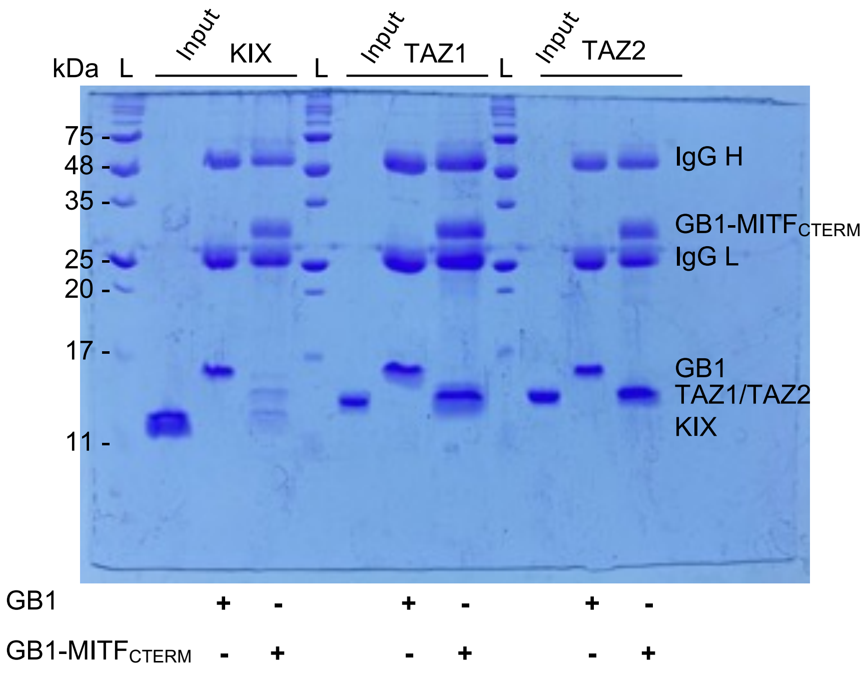

Figure S3. Uncropped gel images of duplicate pulldown experiments shown in Figure 3A. The images are Coomassie-stained SDS-PAGE gels of the total amount of KIX, TAZ1, or TAZ2 pulled down by immobilized GB1 or GB1-MITF$_{CTERM}$. Input lanes represent isolated CBP/p300 domains and are 10% of total pulldown input. The migration of immunoglobulin heavy and light chains (IgG H and IgG L), GB1, GB1-MITF$_{CTERM}$, KIX, TAZ1, and TAZ2 are denoted.

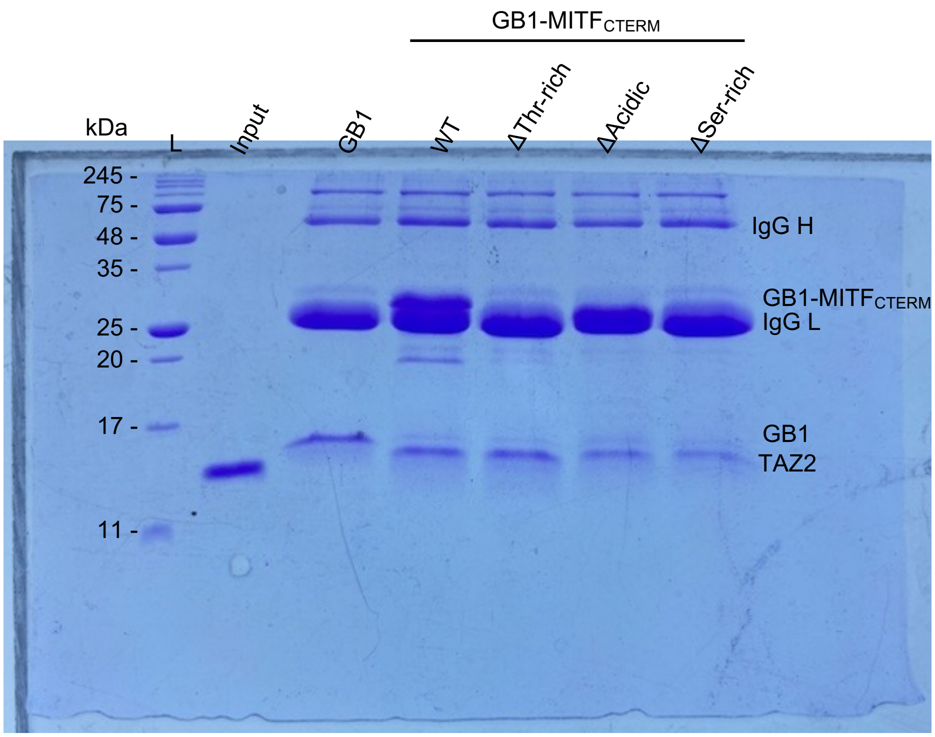

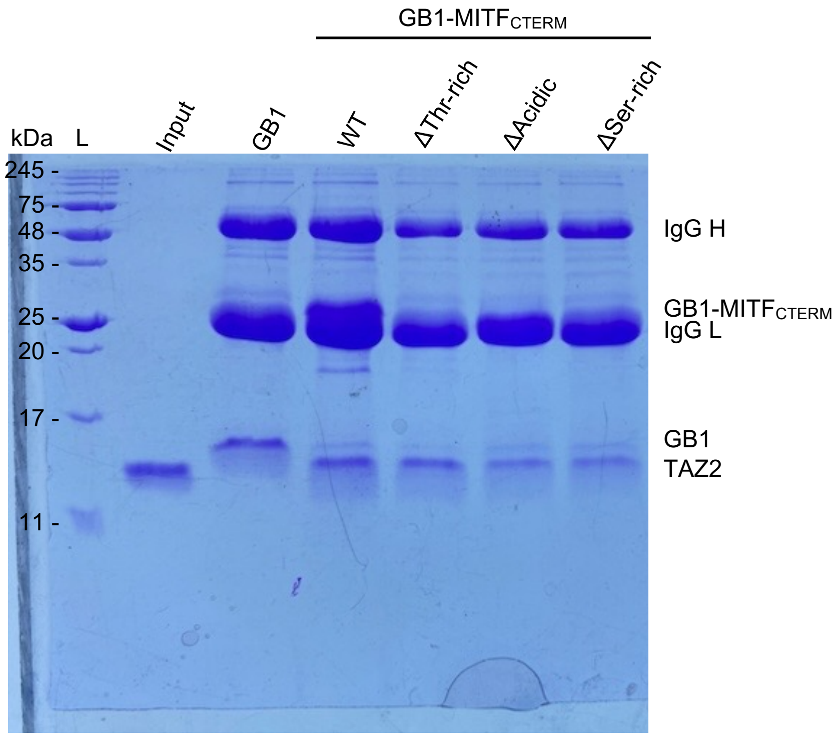

Figure S4. Uncropped gel images of duplicate pulldown experiments shown in Fig. 4C. The images are Coomassie-stained SDS-PAGE gels of the total amount TAZ2 pulled down by GB1 or GB1-MITF$_{CTERM}$ variants. Input lanes represent isolated TAZ2 and are 10% of total pulldown input. The migration of immunoglobulin heavy and light chains (IgG H and IgG L), GB1, GB1-MITF$_{CTERM}$, and TAZ2 are denoted.
